# Quinic and Ferulic Acid–Loaded Chitosan Nanoparticles Attenuate Experimental Ulcerative Colitis by Modulating the IL‐32/IL‐33/IL‐34 Axis and NF‐κB Signaling

**DOI:** 10.1155/ijin/7179301

**Published:** 2026-06-26

**Authors:** Fatemeh Azadegan-Dehkordi, Hossein Aslani, Mehdi Badihi, Fatemeh Taheri, Pegah Khosravian-Dehkordi, Nader Bagheri

**Affiliations:** ^1^ Cellular and Molecular Research Center, Basic Health Sciences Institute, Shahrekord University of Medical Sciences, Shahrekord, Iran, skums.ac.ir; ^2^ Cancer Research Center, Shahrekord University of Medical Sciences, Shahrekord, Iran, skums.ac.ir; ^3^ Student Research Committee, Shahrekord University of Medical Sciences, Shahrekord, Iran, skums.ac.ir; ^4^ Department of Pathology, Shahrekord University of Medical Sciences, Shahrekord, Iran, skums.ac.ir; ^5^ Medical Plants Research Center, Basic Health Sciences Institute, Shahrekord University of Medical Sciences, Shahrekord, Iran, skums.ac.ir; ^6^ Clinical Biochemistry Research Center, Basic Health Sciences Institute, Shahrekord University of Medical Sciences, Shahrekord, Iran, skums.ac.ir

**Keywords:** chitosan nanoparticles, ferulic acid, IL-32/IL-33/IL-34 axis, inflammation, NF-κB pathway, quinic acid, ulcerative colitis

## Abstract

**Introduction:**

Ulcerative colitis (UC) is characterized by immune dysregulation and localized inflammation of the colonic mucosa. Despite advances in therapy, effective treatment remains difficult. This study aimed to investigate the immunomodulatory and therapeutic effects of chitosan nanoparticles (CS‐NPs) loaded with quinic acid (QA) and ferulic acid (FA) in an experimental model of UC, focusing on the IL‐32/IL‐33/IL‐34 cytokine axis and the NF‐κB signaling pathway.

**Methods:**

Eighty male Wistar rats were randomly divided into 10 groups (*n* = 8 per group). Experimental acute colitis was induced in rats via intrarectal administration of 4% acetic acid (AA). After induction, the animals received CS‐NPs loaded with QA and FA for seven consecutive days. IL‐33, IL‐34, and NF‐κB mRNA expression were measured by real‐time PCR. Additionally, tissue IL‐32 levels were measured using an ELISA kit.

**Results:**

Induction of UC significantly increased IL‐32 protein levels as well as the expression of IL‐33, IL‐34, and NF‐κB. Oral administration with mesalazine, FA/QA/CS‐NPs, QA/CS‐NPs, FA/CS‐NPs, FA/QA, QA, and FA significantly decreased IL‐32 protein levels and the expression of IL‐33, IL‐34, and NF‐κB relative to the model group.

**Conclusion:**

FA/QA‐loaded CS‐NPs exhibit anti‐inflammatory effects in experimental UC, likely through modulation of the IL‐32/IL‐33/IL‐34 cytokine axis and NF‐κB signaling. These findings suggest that FA/QA/CS‐NPs may serve as a promising herbal‐based therapeutic candidate for the treatment of UC in humans.

## 1. Introduction

Ulcerative colitis (UC) is a chronic, relapsing inflammatory disease of the colon; affected patients typically exhibit inflammation from the cecum to the rectum [1]. The cause of the disease remains unknown; however, it is believed that UC is a complex disorder involving four main factors: microbiota, mucosal immunology, genetic predisposition, and environmental influences (e.g., medications, diet, and smoking) [1]. Recent evidence indicates that abnormal immune activation and dysfunction are key pathogenic drivers of UC. This condition involves overactive T‐cells, high levels of inflammatory cytokines, an imbalance in gut bacteria, and a weakened intestinal barrier. Overactive T helper 1 (Th1) and Th17 cells play a key role in UC. They produce inflammatory cytokines such as TNF‐α, IL‐17, and IFN‐γ, which lead to ongoing inflammation in the colon.

IL‐32 has emerged as an important player in innate and adaptive immune responses [2]. IL‐32 is produced by a wide range of cells, such as endothelial and epithelial cells, fibroblasts, NK cells, monocytes, and T cells [2, 3]. IL‐32 promotes the transformation of monocytes into macrophages or dendritic cells and triggers the production of inflammatory cytokines such as TNF‐α, IL‐1β, IL‐6, and IL‐8 through p38 mitogen‐activated protein kinase and NF‐κB signaling pathways [4]. IL‐33 belongs to the IL‐1 cytokine family and is produced by various cells, including endothelial and epithelial cells and keratinocytes, as well as astrocytes, dendritic cells, and monocytes [5]. IL‐33 levels are increased in several autoimmune diseases [5]. IL‐34 binds to the same receptor as macrophage colony‐stimulating factor (M‐CSF), known as CSF‐1R, and promotes lymphocyte differentiation and proliferation while regulating the production of inflammatory molecules. Recent studies have shown abnormal IL‐34 expression in several autoimmune diseases [6].

Quinic acid (QA), a phenolic compound extracted from various plants, including cinchona bark, coffee beans, and certain fruits and vegetables, exhibits anti‐inflammatory, antioxidant, and antiapoptotic properties [7]. Ferulic acid (FA) is a natural compound found in vegetables and in the seeds and cell walls of grains like rice and oats. It has been shown to have anti‐inflammatory, antioxidant, antidiabetic, and anticancer activities [8]. Our recent studies showed that chitosan nanoparticles (CS‐NPs) loaded with QA and FA improved colitis in rats by increasing colon length and reducing tissue damage, DAI score, Th17 cells, and inflammatory cytokines (IL‐6, IL‐23, IL‐17A, TGF‐β, TNF‐α, IL‐1β, IL‐8, and IL‐36α) [9].

Given the beneficial effects of QA and FA in UC, further studies are needed to better understand their therapeutic potential and clarify how they act on other inflammatory and oxidative pathways involved in the disease. Therefore, this study aimed to evaluate the effects of QA, FA, and pH‐sensitive NPS in a rat model of acetic acid (AA)‐induced UC by assessing tissue levels of IL‐33, IL‐34, NF‐κB gene expression, and the proinflammatory cytokine IL‐32 protein.

## 2. Materials and Methods

### 2.1. Animals

Eighty healthy adult male Wistar rats (8–10 weeks old, 220–240 g) were housed under standard laboratory conditions (22 ± 2°C; 12/12 h light/dark cycle) with free access to food and water.

### 2.2. Drugs and Chemicals

FA (CAS 537‐98‐4, ≥ 99% HPLC) and QA (CAS 77‐95‐2, ≥ 98% HPLC) were purchased from Sigma‐Aldrich. All other compounds were of the highest analytical grade and purity.

### 2.3. Study Design and Treatments

In this study, we had 80 male rats. We split them into 10 groups, and every group had eight rats. Treatment details are shown in Table 1. All drugs were given by gastric gavage once daily for 7 days, starting 1 h after colitis induction. Dosages were determined using a pilot study and prior published data [7, 10]. On Day 8, rats were deeply anesthetized with intraperitoneal ketamine (100 mg/kg) and xylazine (10 mg/kg), then euthanized by guillotine decapitation following AVMA (2020) and NIH (8th ed., 2011) guidelines. The colon was excised, washed with phosphate‐buffered saline, and frozen at −80°C.

**TABLE 1:** Experimental grouping and treatment regimen used in the study.

Group	Description/condition	Treatment and dose	Route/duration
Group I	Sham Control	Normal saline (1 mL)	Intrarectal, single dose
Group II	AA‐induced colitis (model)	Acetic acid 4% (1 mL)	Intrarectal, single dose
Group III	AA + mesalazine	Mesalazine (300 mg/kg/day)	Oral gavage, 7 days
Group IV	AA + CS‐NPs	CS‐NPs (no drug)	Oral gavage, 7 days
Group V	AA + FA	FA (60 mg/kg/day)	Oral gavage, 7 days
Group VI	AA + FA‐loaded NPs	CS‐NPs + FA (60 mg/kg/day)	Oral gavage, 7 days
Group VII	AA + QA	QA (100 mg/kg/day)	Oral gavage, 7 days
Group VIII	AA + QA‐loaded NPs	CS‐NPs + QA (100 mg/kg/day)	Oral gavage, 7 days
Group IX	AA + QA + FA	QA (100 mg/kg/day) + FA (60 mg/kg/day)	Oral gavage, 7 days
Group X	AA + QA + FA‐loaded NPs	CS‐NPs + QA (100 mg/kg/day) + FA (60 mg/kg/day)	Oral gavage, 7 days

### 2.4. Preparation of QA‐ and FA–Loaded CS‐NPs

QA‐ and FA–loaded CS‐NPs were prepared using the ionic gelation method as previously described in our earlier study [9]. For nanoparticle preparation, 60 mg of chitosan was stirred in 20 mL of 0.1 M AA for 4 h. This solution was then mixed with either plain ethanol or ethanol containing FA (600 mg), QA (1000 mg), or both, resulting in empty, FA‐loaded, QA‐loaded, and dual‐loaded nanoparticles, respectively. The mixture was kept stirring at 500 rpm for 2 h (24°C, protected from light). Subsequently, a solution of 20 mg of TPP in 20 mL of water was introduced dropwise. After another 2 h of stirring, the mixture was sonicated for 20 min and stirred again. Then, a solution containing 20 mg of EU S100 dissolved in 5 mL of acetone was introduced dropwise. The mixture was then placed on a rotator at room temperature for 24 h to facilitate acetone evaporation. There was too much ethanol in the mixture. So we evaporated the extra ethanol until the liquid volume went down to 40 mL. Final drug concentrations were 0, 15 mg/mL FA, 25 mg/mL QA, or 15 mg/mL FA + 25 mg/mL QA. The detailed preparation procedure and physicochemical characterization of the NPs, including zeta potential, drug loading efficiency, and particle size, have been reported previously [9].

### 2.5. Physicochemical Characterization of NPs

The physicochemical properties of QA‐ and FA–loaded chitosan NPs, including particle size distribution, zeta potential, and morphology, were previously characterized in detail in our earlier study [9]. Particle size and polydispersity index were both measured using dynamic light scattering (DLS). Zeta potential was subsequently measured using a zeta potential analyzer. Field emission scanning electron microscopy (FE‐SEM) analysis was used to evaluate the morphological features of the NPs. The findings confirmed both their spherical geometry and nanoscale dimensions. Detailed characterization data have been reported previously [9].

### 2.6. Gene Expression Analysis by Real‐Time Quantitative PCR (qPCR)

To measure gene expression, we first isolated total RNA from colon tissue using a commercial kit (RNX‐Plus Solution). A NanoDrop device checked the RNA’s purity and concentration. Then, we converted 4 μg of RNA into cDNA using another kit. This cDNA was amplified in a real‐time PCR machine with SYBR Green dye. The machine was programmed to heat the samples to 95°C first, then repeat a three‐step cycle 40 times: 95°C (10 s), 60°C (15 s), and 72°C (20 s). A melting curve test confirmed we only amplified the right genes. The expression levels of IL‐33, IL‐34, and NF‐κB were compared to a control gene (β‐actin) and calculated using a standard formula (2^−ΔΔCt^) [11]. We have listed all the qPCR primer sequences in Table 2.

**TABLE 2:** Primer sequences.

Gene	Primer sequence
β‐Actin	Forward: 5‐GTCAGGTCATCACTATCGGCAAT‐3
	Reverse: 5‐AGAGGTCTTTACGGATGTCAACGT‐3

NF‐κB	Forward: 5‐CATTAATATTTAACGATGTGGATGCGTTTCA‐3
	Reverse: 5‐ GCCTACCATCTTTAAACTGCACAAT‐3

IL‐33	Forward: 5‐ACTGCTAACAGAATCTTGTGCC‐3
	Reverse: 5‐ATCCACACCGTCTCCTGATTG‐3

IL‐34	Forward: 5‐CGCCTGGTTATACTGTCTTGG‐3
	Reverse: 5‐ACTCTGAGTACCCCCTCATAAG‐3

### 2.7. Evaluating the Inflammatory Protein IL‐32 in the Colon

Colon samples were first homogenized in ice‐cold RIPA lysis buffer that contained 1% protease inhibitor cocktail. The homogenate was then centrifuged at 13,000 rpm for 20 min at 4°C, and the resulting supernatant was stored at −80°C until it was used for analysis. IL‐32 protein levels in the samples were subsequently measured using commercial ELISA kits (ZellBio, Germany), following the instructions provided by the manufacturer.

### 2.8. Data Analysis

We used GraphPad Prism 8.4.3 software for all statistical calculations. The data are shown as average values plus or minus the standard error of the mean (mean ± SEM). First, we checked if the data followed a normal distribution using the Kolmogorov–Smirnov test. Then, we compared the different groups using one‐way ANOVA, and for more detailed comparisons between specific groups, we used Tukey’s post hoc test. We considered a *p* value of 0.05 or lower to be statistically significant.

## 3. Results

### 3.1. Evaluation of NPs Characteristics

#### 3.1.1. Particle Size and Zeta Potential Analysis

These results were adapted from our previous study [9]. The detailed size and zeta potential measurements of the prepared nanoparticles are presented in Table 3. As shown, all formulations exhibited appropriate hydrodynamic diameters and positive surface charges, indicating good colloidal stability. These physicochemical properties confirmed that the nanoparticles were suitable for oral administration in subsequent in vivo experiments.

**TABLE 3:** Particle size and zeta potential of nanoformulations (adapted from [9]).

Nanoparticle formulation	Hydrodynamic size (nm)	Polydispersity index (PDI)	Zeta potential (mV)
CS‐TPP/EU S100 (empty)	99.8 ± 3.6	0.21–0.33	+31.1
FA/CS‐TPP/EU S100	109.7 ± 4.2	0.21–0.33	+29.9
QA/CS‐TPP/EU S100	170.6 ± 6.1	0.21–0.33	+31.4
FA/QA/CS‐TPP/EU S100	117.1 ± 5.0	0.21–0.33	+23.8

#### 3.1.2. SEM Morphological Assessment

As reported by Bedahi et al. [9], FE‐SEM imaging revealed irregular spherical morphology across all nanoparticle formulations. Empty CS‐TPP/EU S100 NPs exhibited sizes of 70–88 nm. Drug loading and Eudragit coating increased particle size ranges to 85–104 nm (FA‐loaded), 78–121 nm (QA‐loaded), and 98–149 nm (FA/QA‐loaded).

### 3.2. Effects of Mesalazine and FA/QA Nanoformulations on IL‐32 Protein Levels in the Colon

**FIGURE 1:**
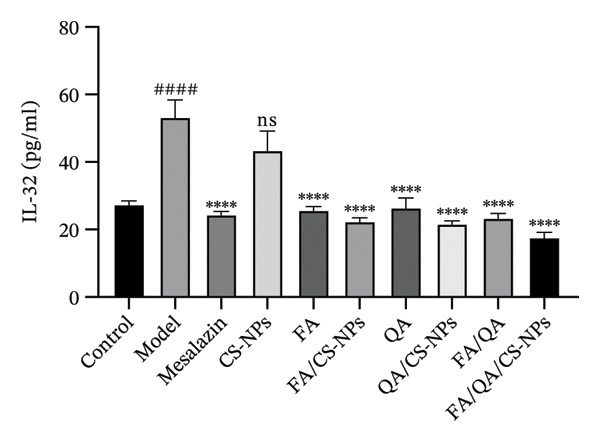
Effects of different groups on the protein levels of IL‐32 in the colons of rats with AA‐induced UC. Data represent the mean ± SEM. ns: nonsignificant differences. ^###^ *p* < 0.001, ^####^ *p* < 0.0001 compared to the control group. ^∗^ *p* < 0.05, ^∗∗^ *p* < 0.01, ^∗∗∗^ *p* < 0.001, ^∗∗∗∗^ *p* < 0.0001 compared to the UC group. There was a significant difference between groups using the one‐way ANOVA and Tukey post hoc test multiple comparison analysis.

Proinflammatory cytokines such as IL‐32 are important indicators of inflammatory diseases. We therefore used ELISA to measure IL‐32 protein levels following treatment with mesalazine and various FA/QA nanoformulations. IL‐32 was significantly elevated in the model group versus controls (Figure 1). All oral treatments significantly reduced IL‐32 compared to the model group (Figure 1). The rank order of efficacy (greatest to least reduction) was: FA/QA/CS‐NPs > QA/CS‐NPs > FA/CS‐NPs > FA/QA > mesalazine > FA > QA.

### 3.3. Effects of Mesalazine and FA/QA Nanoformulations on NF‐κB, IL‐33, and IL‐34 mRNA Expression Levels in the Colon

**FIGURE 2:**
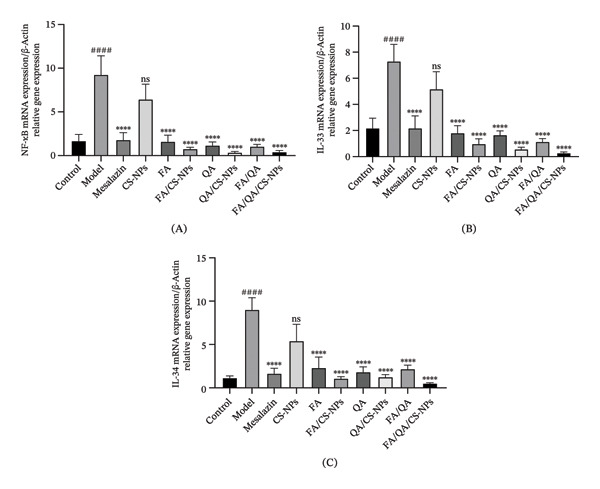
Effects of different groups on the expression of NF‐κB (A), IL‐33 (B), and IL‐34 (C) mRNA in the colons of rats with AA‐induced UC. Data represent the mean ± SEM. ns: nonsignificant differences. ^###^ *p* < 0.001, ^####^ *p* < 0.0001 compared to the control group. ^∗^ *p* < 0.05, ^∗∗^ *p* < 0.01, ^∗∗∗^ *p* < 0.001, ^∗∗∗∗^ *p* < 0.0001 compared to the UC group. There was a significant difference between groups using the one‐way ANOVA and Tukey post hoc test multiple comparison analysis.

Following AA administration, mRNA expression levels of NF‐κB, IL‐33, and IL‐34 in colon tissue were significantly elevated compared to controls. As shown in Figure 2A–D, the model group exhibited marked increases in NF‐κB (fold change [FC] = 5.48, *p* < 0.0001), IL‐33 (FC = 3.35, *p* < 0.0001), and IL‐34 (FC = 7.78, *p* < 0.0001) relative to the control group. Oral administration with mesalazine, free FA, FA/CS‐NPs, free QA, QA/CS‐NPs, the free FA/QA combination, and FA/QA/CS‐NPs significantly reduced NF‐κB mRNA expression compared to the model group, with FCs of −5.12, −5.75, −11.8, −7.9, −14.15, −8.78, and −21.55, respectively (all *p* < 0.0001; Figure 2A). Similarly, all treatments significantly lowered IL‐33 mRNA expression compared to the UC group. The FCs were as follows: mesalazine (−3.5), CS‐NPs (−4), free FA (−7.45), FA/CS‐NPs (−4.5), free QA (−12.6), QA/CS‐NPs (−6.35), FA/QA (−25.8), and FA/QA/CS‐NPs (Figure 2B). All reductions were statistically significant (*p* < 0.0001). IL‐34 mRNA expression was also significantly decreased by all treatments compared to the UC group. FCs were mesalazine (−5.6), CS‐NPs (−3.9), free FA (−8.3), FA/CS‐NPs (−5), free QA (−7.5), QA/CS‐NPs (−4.3), and FA/QA/CS‐NPs (−17) (all *p* < 0.0001; Figure 2C). These findings indicate that the NPs effectively suppress key proinflammatory mediators, highlighting their potential therapeutic advantage over free compounds in mitigating colonic inflammation.

## 4. Discussion

UC, the most common form of IBD, features recurrent mucosal inflammation of the colon [1, 12]. The exact etiology of UC remains unclear. Nevertheless, current evidence suggests that intestinal immune dysregulation and excessive proinflammatory cytokine secretion are key drivers of mucosal barrier damage and persistent inflammation [13]. Accumulation of various immune cells and inflammatory cytokines within the large intestine leads to aberrant immune responses [14]. Sulfasalazine and mesalazine are widely used anti‐inflammatory drugs for UC treatment [15]. Adverse effects of these drugs include toxicity, vomiting, anemia, and generalized edema. Thus, traditional herbs have gained popularity due to their abundant natural compounds. These compounds exhibit anti‐inflammatory properties, making them valuable for treating inflammatory diseases [16].

Our recent studies demonstrated that QA‐ and FA–loaded CS‐NPs exhibited suitable size and zeta potential. FTIR analysis confirmed successful drug loading onto the NPs without significant structural alterations. No drug release occurred in simulated gastric fluid (SGF), whereas both drugs showed a slow‐release profile in simulated intestinal fluid (SIF). Intrarectal AA (1 mL, 4%) increased the frequency of both Th1 and Th17 immune cells, upregulated IL‐6, IL‐23, IL‐17A, and TGF‐β, and raised IL‐1β, IL‐8, IL‐36α, and TNF‐α protein levels. UC also reduced colon length and elevated macroscopic damage, DAI score, colon weight, and histological damage score [9, 17]. Our findings demonstrated that intrarectal AA (1 mL, 4%) elevated IL‐32 protein levels and upregulated IL‐33, IL‐34, and NF‐κB expression.

Shioya et al. reported a significant increase in epithelial IL‐32α expression in the inflamed mucosa of IBD patients, particularly those with Crohn’s disease, compared with normal tissues. They also showed that proinflammatory cytokines upregulated IL‐32α expression via NF‐κB activation, indicating a key role for IL‐32α in IBD‐related inflammatory responses [18]. IL‐32 is a proinflammatory cytokine that induces the release of IL‐1β, IL‐6, TNF‐α, and chemokines via activation of the p38 MAPK and NF‐κB pathways [2, 19]. These findings suggest that increased IL‐32 secretion by intestinal epithelial cells stimulates infiltrating immune cells to produce proinflammatory cytokines, thereby exacerbating mucosal inflammation. Our study showed that oral administration of FA/QA/CS‐NPs, QA/CS‐NPs, FA/CS‐NPs, FA/QA, QA, FA, and mesalazine significantly decreased IL‐32 protein and NF‐κB expression. FA/QA/CS‐NPs exhibited the greatest reduction in both parameters.

Gundersen et al. reported that IL‐33 expression is markedly increased in the colonic crypt epithelium during active UC but significantly reduced or absent during remission. Loss of epithelial IL‐33 was strongly associated with mucosal healing and clinical improvement, and its expression was downregulated following anti‐TNF therapy, further supporting its potential as a biomarker of disease activity and therapeutic response. These findings highlight the important role of IL‐33 in intestinal inflammation and epithelial immune regulation [20]. Our study showed that oral administration of FA/QA/CS‐NPs, QA/CS‐NPs, FA/CS‐NPs, FA/QA, QA, FA, and mesalazine significantly decreased IL‐33 expression. FA/QA/CS‐NPs showed the greatest reduction in IL‐33 levels.

Zwicker et al. reported that IL‐34 expression is increased in inflamed tissues of IBD patients and experimental colitis, particularly in intestinal epithelial cells and infiltrating lamina propria cells. They further showed that blocking the NF‐κB pathway reduced TNF‐α‐stimulated IL‐34 expression in colon epithelial cells, suggesting that macrophage‐derived TNF‐α may induce IL‐34 via NF‐κB signaling [21]. In contrast to our findings, Chen et al. reported that upregulation of IL‐34 using genetically engineered bacteria alleviated colitis and improved intestinal barrier function. Their results showed increased expression of tight junction proteins such as ZO‐1 and occludin, suggesting a protective role of IL‐34 in maintaining mucosal integrity [22]. Similarly, Liu et al. demonstrated that IL‐34 deficiency exacerbates experimental colitis and promotes inflammation‐associated tumor development. IL‐34 knockout mice showed increased inflammation, epithelial damage, and impaired mucosal barrier function, further supporting a protective role of IL‐34 in intestinal homeostasis [23]. Our study showed that oral administration of FA/QA/CS‐NPs, QA/CS‐NPs, FA/CS‐NPs, FA/QA, QA, FA, and mesalazine significantly decreased IL‐34 expression. FA/QA/CS‐NPs showed the greatest reduction in IL‐34 levels. Taken together, these findings indicate that IL‐34 may exert context‐dependent effects in UC. While IL‐34 can support epithelial integrity and limit excessive inflammation under certain conditions, its elevated expression in inflamed tissues may also reflect ongoing immune activation. Therefore, the downregulation of IL‐34 observed in our study likely reflects an overall reduction in inflammatory responses rather than a direct inhibition of its protective functions.

The FA/QA‐loaded chitosan NPs showed stronger anti‐inflammatory effects than either compound alone, suggesting a synergistic interaction. FA provides antioxidant properties, while QA modulates inflammatory pathways. Their co‐delivery enhances suppression of mediators like IL‐32/IL‐33/IL‐34 cytokine axis and NF‐κB signaling. In addition, the use of chitosan‐based NPs offers several advantages over free compounds, including improved drug stability, protection from premature degradation, enhanced bioavailability, and more efficient delivery to inflamed colonic tissues. These features may explain the superior therapeutic effects observed for the nanoformulations, particularly the FA/QA combination. Furthermore, previous studies have demonstrated that nanoparticle‐based delivery systems can significantly improve treatment outcomes in experimental colitis by enhancing drug accumulation at inflamed sites and reducing systemic side effects [24, 25]. In this context, the findings of the present study are consistent with and extend previous reports, highlighting the potential of combined nano‐delivery strategies using natural anti‐inflammatory compounds for the management of UC.

Oxidative stress and excessive production of reactive oxygen species (ROS) play a critical role in the pathogenesis and progression of UC [26]. Although ROS levels were not directly measured in the present study, both FA and QA are well‐documented for their potent antioxidant and ROS‐scavenging properties. A recent study by Ekhtiar et al. [27] demonstrated that treatment with FA and QA significantly upregulated the expression of Nrf2, HO‐1, and NQO1, key regulators of the antioxidant response, while reducing proinflammatory cytokines TNF‐α and IL‐1β in a rat model of AA‐induced colitis. Importantly, the study concluded that the anti‐inflammatory effects of FA and QA are mediated at least in part through activation of the Nrf2/HO‐1 signaling pathway [27]. Ghasemi‐Dehnoo et al. demonstrated that FA and QA significantly increased the activity of antioxidant factors, including total antioxidant capacity (TAC), superoxide dismutase (SOD), and catalase (CAT), while reducing malondialdehyde (MDA) and nitric oxide (NO) levels in a rat model of colitis. Additionally, FA has been shown to inhibit inflammatory signaling pathways such as TLR4/NF‐κB [7, 10]. Collectively, these findings strongly suggest that the therapeutic effects observed with FA/QA/CS‐TPP/EU S100 NPs in the present study may be partially attributed to ROS scavenging and subsequent modulation of inflammatory signaling pathways. Future studies directly measuring intracellular ROS levels and assessing Nrf2/HO‐1 pathway activation are warranted to confirm this mechanism.

The main limitation of this study is the lack of a comprehensive assessment of the systemic toxicity of the synthesized NPs. In addition, the cellular activity and uptake of QA‐ and FA‐loaded CS‐NPs were not evaluated using in vitro cell models. Evaluating the effects of these NPs on intestinal epithelial cells or immune cells could provide deeper insight into their anti‐inflammatory mechanisms. Furthermore, only a single experimental model of UC was used in this study. Future investigations should focus on clarifying the molecular mechanisms of NP‐based formulations, assessing their cellular interactions, and establishing standardized criteria for evaluating their therapeutic efficacy in UC.

## 5. Conclusions

In conclusion, our study demonstrates that FA‐ and QA–loaded CS‐NPs effectively reduce inflammation in experimental colitis. Treatment, particularly with FA/QA/CS‐NPs, reduced the IL‐32/IL‐33/IL‐34 cytokine axis and NF‐κB signaling, highlighting their potential as a promising strategy for managing UC.

## Author Contributions

Fatemeh Azadegan‐Dehkordi: conceptualization, data curation, methodology, investigation, resources, and writing–original draft. Hossein Aslani: data curation, methodology, writing–original draft, and investigation. Mehdi Badihi: methodology, investigation, and resources. Fatemeh Taheri: data curation, methodology, and investigation. Pegah Khosravian‐Dehkordi: methodology, data curation, investigation, resources, and writing–original draft. Nader Bagheri: formal analysis, validation, investigation, project administration, supervision, resources, funding acquisition, and writing–review and editing.

## Funding

This study was supported by the Shahrekord University of Medical Sciences (grant number: 4537).

## Ethics Statement

This study was approved by the Ethics Committee of Shahrekord University of Medical Sciences, Shahrekord, Iran (IR.SKUMS.AEC.1403.021).

## Conflicts of Interest

The authors declare no conflicts of interest.

## Data Availability

The supporting data for this study are available from the corresponding author upon reasonable request.
